# Research progress on the role of host factors in Chikungunya virus infection and intervention strategies

**DOI:** 10.1080/21505594.2026.2679253

**Published:** 2026-05-27

**Authors:** Yibo Chen, Ke Zhang, Zhongtian Qi, Cuiling Ding

**Affiliations:** a Department of Microbiology, Shanghai Key Laboratory of Medical Bioprotection, Key Laboratory of Biological Defense, Ministry of Education, Naval Medical University, Shanghai, China; b Independent Researcher, Shanghai, China

**Keywords:** Chikungunya virus, host factors, virus-host interaction, innate immunity, antiviral drugs targeting host factors

## Abstract

Chikungunya virus (CHIKV) is a mosquito-borne alphavirus causing acute fever, rashes, and even acute or chronic arthralgia/arthritis. With its expanding global distribution, CHIKV represents a significant public health threat, yet no specific antivirals are approved. The lifecycle of CHIKV is highly dependent on interactions with host factors that mediate viral entry, genome replication, protein translation, assembly, and release. A deeper understanding of these host factors provides new insights into CHIKV pathogenesis and offers strategies to overcome drug resistance associated with high viral mutation rates. This review summarizes and updates current knowledge on the roles of host factors in CHIKV infection, systematically dissects the interaction network and cell-type specificity of key host factors, critically evaluates the druggability of host-directed therapeutic targets, and outlines recent progress in anti-CHIKV drug development, with a focus on strategies targeting both viral proteins and host dependencies, placing a particular emphasis on host-directed antiviral therapies.

## Introduction

Chikungunya virus (CHIKV) is a small (approximately 70 nm in diameter) enveloped, positive-sense RNA virus, belonging to *Alphavirus* genus, *Togaviridae* family [1]. It causes chikungunya fever (CHIKF), an acute infection characterized by fever, rashes, arthralgia and frequent progression to chronic joint symptoms. Although the case fatality rate is low (~0.1–0.13%), approximately 50% of patients develop persistent arthralgia/arthritis lasting months to years, severely affecting quality of life [2]. Phylogenetic analyses classify CHIKV into three major genotypes: West African (WA), East/Central/South African (ECSA), and Asian. Currently, the ECSA and Asian genotypes are the most prevalent [3]. First isolated during a 1952 outbreak in Tanzania, CHIKV circulated mainly in Africa and Asia over subsequent decades [4]. Since the early twenty-first century, however, its geographic range has broadened considerably. In the past 20 y, CHIKV has caused over 10 million cases across more than 125 countries or regions [5]. The primary vectors are *Aedes aegypti* and *Aedes albopictus* for CHIKV. Before 2004, transmission relied mainly on *Aedes aegypti* [6]. During a 2004–2005 outbreak in Kenya, an alanine to valine substitution at position 226 of the E1 structural protein (E1:A226V) emerged in the ECSA genotype, forming the Indian Ocean lineage (IOL) [4]. This mutation significantly enhanced the adaptation to *Aedes albopictus*, facilitating the spread of CHIKV into temperate zones [7]. Reflecting its growing threat, CHIKV was added to the WHO priority pathogens list in May 2024 [8]. According to the European Centre for Disease Prevention and Control, from January to July 2025, approximately 240,000 cases of CHIKF and 90 CHIKF-related deaths were reported across 16 countries and regions worldwide, with Asia alone accounting for over 34,000 cases [9]. A recent outbreak in Guangdong Province, China – with over 13,000 cases reported since July 2025 (as of October 11) – underscores how ecological changes and global connectivity amplify infectious disease risks [10].

The U.S. Food and Drug Administration (FDA) approved the first live-attenuated CHIKV vaccine, Ixchiq, on 9 November 2023 [11]; however, the vaccine was halted on 22 August 2025, due to “serious safety concerns.” On 14 February 2025, a virus-like particle-based vaccine named VIMKUNYA was approved by FDA [12]. Despite these advances, no specific antiviral drug against CHIKV is yet available. Developing novel antiviral strategies requires a deep understanding of viral infection mechanisms and CHIKV host interactions. Existing reviews have mostly focused on the individual function of single host factors in CHIKV infection [13,14], while a systematic dissection of the crosstalk between host factors, their cell-type specific roles in viral tropism, and the critical regulatory mechanisms of immune inflammation and chronic disease remains insufficient. Therefore, further investigation into CHIKV infection and therapeutic development holds significant strategic importance. This review focuses on CHIKV genome structure, the viral lifecycle, host factors involved in infection, the regulatory network of host factors in immune inflammation and chronic infection, their cell-type specificity, and the crosstalk between different host factors, and recent advances in antiviral drug research with a critical analysis of host-targeted therapeutic strategies.

## Genome structure and life cycle of CHIKV

The CHIKV genome RNA comprises approximately 11,800 nucleotides and contains two open reading frames (ORFs) flanked by 5’and 3’ untranslated regions. The 5” ORF encodes four non-structural proteins (nsP1-4) primarily involved in intracellular viral replication. The 3” ORF is transcribed into a subgenomic RNA that encodes six structural proteins: capsid (C), E3, E2, 6K, transframe (TF), and E1. C, E1, and E2 are the major structural proteins that, together with the genomic RNA, constitute the virion.

CHIKV non-structural proteins (nsPs) mediate viral RNA replication and transcription while hijacking host cell machinery. NsP1, a membrane anchored component of the replication complex, caps the 5’ end of viral RNA via an unconventional mechanism, protecting the RNA and enabling efficient translation [15,16]. NsP2 is a multifunctional protein exhibiting helicase, NTPase, RNA triphosphatase, and protease activities [17,18]. It processes viral polyprotein precursors and participates in RNA replication [19]; it can also enter the nucleus to suppress host transcription and translation [20]. NsP3 acts as a scaffold for the viral replication complex [21,22]. Its N-terminal macrodomain hydrolyzes ADP ribose modifications on host proteins to counteract antiviral signaling [23], while the C-terminal region recruits host proteins such as G3BP to suppress stress granule formation and establish viral replication factories [21]. NsP4 functions as the RNA dependent RNA polymerase (RdRp), the core enzyme for viral RNA synthesis [24].

The structural proteins (sPs) form the viral particle and mediate genome protection, cell entry, immunogenicity, and assembly [25]. The ~30 kDa capsid (C) protein contains domains responsible for nonspecific RNA binding, specific genome recognition, and serine protease activity [26,27]. It plays a central role in genome packaging, capsid assembly, and viral maturation, and its nucleocytoplasmic shuttling is essential for infection [28]. E3 acts as a chaperone for E2 folding and stabilization [25,29]. Although E3 dissociates from E2 in the Golgi, its retention on viral particles prevents premature activation of envelope glycoproteins at neutral pH [30]. E2 serves as the primary receptor binding protein and major antigen [27], mediating attachment to host cells via multiple factors including matrix remodeling associated protein 8, glycosaminoglycans, and phosphatidylserine receptors [31–33]. The small TF/6K proteins, produced by programmed frameshifting, are thought to promote viral budding by modulating membrane permeability or forming ion channels, thereby contributing to particle stability and infectivity [26]. E1 is a class II fusion protein that mediates viral endosomal membrane fusion within the acidic environment of endosomes after entry [34,35]. Key mutations in E1 (e.g. E1:A226V) can dramatically alter viral transmissibility and host range, impacting epidemic potential [4].

**Figure 1.**
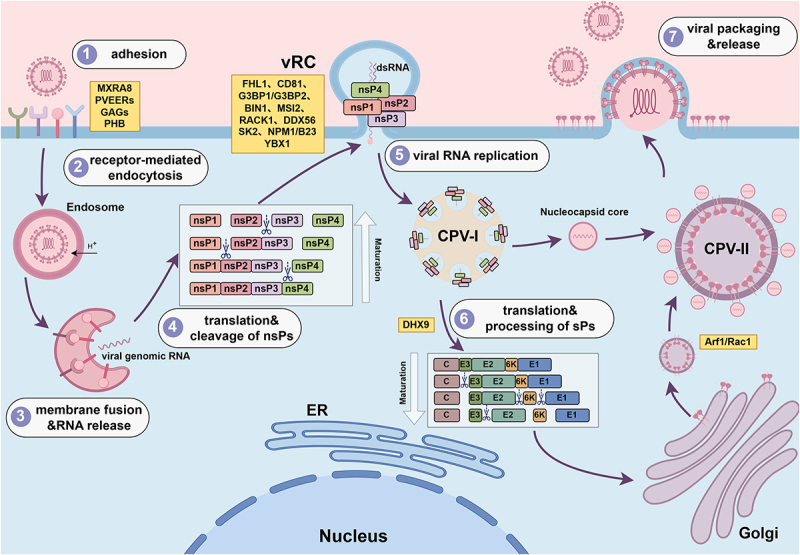
CHIKV life cycle and critical host factors.

The CHIKV envelope displays 80 trimeric spikes composed of E2-E1 heterodimers. E2 mediates receptor binding, while E1 drives membrane fusion [36,37]. The life cycle of CHIKV infection in target cells involves seven key steps: ① Viral attachment; ② Receptor-mediated endocytosis; ③ Membrane fusion and RNA release; ④ Translation and processing of non-structural proteins; ⑤ Viral RNA replication; ⑥ Translation and processing of structural proteins; ⑦ Viral packaging and release (Figure 1).

Following attachment via E2, CHIKV enters cells through receptor mediated endocytosis [38]. Acidification within the endosome triggers conformational rearrangement of the E1/E2 heterodimer, leading to viral endosomal membrane fusion. The nucleocapsid disassembles in the cytoplasm, releasing genomic RNA that is directly translated into P123 and P1234 polyprotein precursors [37]. The P1234 polyprotein is rapidly cleaved by nsP2 protease at the nsP3/nsP4 junction, releasing active nsP4 (Figure 1). P123 and nsP4, together with viral RNA, are recruited to the plasma membrane where they assemble with host factors into an early replication complex. This complex induces the formation of spherical, membrane bound vesicles termed replication spherules or viral replication complexes (vRCs). Within vRCs, CHIKV RNA is replicated via a double stranded RNA (dsRNA) intermediate. Following internalization, vRCs fuse with endosomes to form large (600–2000 nm) vacuolar structures known as type I cytopathic vacuoles (CPV I) [37,39] (Figure 1). Newly synthesized RNA exits CPV I through a pore and diffuses into the cytoplasm, where subgenomic RNA is translated into the structural polyprotein (C-E3-E2-6K-E1). Autocatalytic cleavage by the C protein releases mature C, which oligomerizes with newly synthesized genomic RNA to form nucleocapsids. The remaining polyprotein (E3-E2-6K-E1) is inserted into the endoplasmic reticulum (ER) membrane for processing. Host signal peptidases cleave the 6K protein, allowing E1, E2, and E3 to assemble into immature trimers. These complexes traffic through the secretory pathway, undergoing conformational changes and post translational modifications (palmitoylation, N-linked glycosylation) [37]. Mature E glycoproteins assemble in the Golgi and are transported to the plasma membrane of type II cytopathic vacuoles (CPV II), where they interact with cytoplasmic nucleocapsids. At the plasma membrane, the E2 cytoplasmic domain engages the hydrophobic region of C protein, ultimately driving budding and release of progeny virions [37] (Figure 1).

Although the CHIKV lifecycle is relatively well characterized, molecular targets for antiviral intervention remain limited. Further research is needed to clarify the precise roles of CHIKV proteins in infection and pathogenesis, as well as the key host factors that regulate the viral lifecycle and their synergistic regulatory network.

## Key host factors directly influencing the CHIKV life cycle

CHIKV invasion and replication depend on numerous host cell factors, whose expression or function directly modulates the viral lifecycle (Supplementary table 1).

### Host factors affecting CHIKV entry

Viral entry relies on adhesion factors and specific receptors on the host cell surface. Adhesion factors mediate initial attachment, while receptors facilitate internalization. Key host molecules involved in CHIKV binding and entry include matrix remodeling associated protein 8 (MXRA8), phosphatidylserine mediated virus entry enhancing receptors (PVEERs), glycosaminoglycans (GAGs), prohibitin (PHB), as well as pattern recognition receptors DC-SIGN/L-SIGN and TLR4.

#### Matrix remodeling associated protein 8 (MXRA8)

MXRA8 is widely recognized as a functional receptor for multiple alphaviruses and is expressed predominantly on fibroblasts, osteoblasts, chondrocytes, and skeletal muscle cells. Overexpression of MXRA8 in non-permissive cells enhances CHIKV infection, while its knockout significantly reduces infection by CHIKV and related alphaviruses such as O’nyong’nyong virus (ONNV), Mayaro virus (MAYV), Ross River virus (RRV), and Semliki Forest virus (SFV) [32]. Blocking MXRA8 with monoclonal antibodies or soluble MXRA8 Fc fusion proteins inhibits CHIKV infection and reduces pathogenicity in cellular and mouse models [32,40]. Structural studies show that a single MXRA8 molecule binds E2 E1 heterodimers within the same viral spike [34]. Notably, different CHIKV strains exhibit varying dependence on MXRA8, and not all target cells express it [32], indicating that MXRA8 is not the sole receptor for CHIKV.

#### Phosphatidylserine-mediated virus entry enhancing receptors (PVEERs)

PVEERs are receptors or complexes that enhance attachment and endocytosis of enveloped viruses. T cell immunoglobulin and mucin domain protein 1 (TIM-1) and Axl receptor tyrosine kinase are PVEERs that promote entry of alphaviruses, filoviruses, and flaviviruses [41]. The TIM protein family and Axl have been shown to enhance CHIKV pseudovirus infection [42]. Axl binds phosphatidylserine indirectly via its ligands growth arrest specific factor 6 (Gas6) [43] or protein S1 (ProS1) [44]. TIM-1 overexpression in HEK293T cells increases CHIKV binding and endocytosis, while TIM-1 specific antibodies reduce infection in a dose dependent manner [41]. TIM-1 and Axl are mainly expressed in macrophages and renal epithelial cells [45] and are the key host factors mediating CHIKV entry in these cell types [14], which is an important supplement to MXRA8-mediated entry pathway.

#### Glycosaminoglycans (GAGs)

GAGs are negatively charged polysaccharides present in the extracellular matrix that serve as universal attachment factors for many arboviruses. Highly sulfated GAGs, such as heparan sulfate (HS) and chondroitin sulfate/dermatan sulfate, promote CHIKV infection [46]. Serial passage of wild type CHIKV in HS rich HEK 293T cells selected for adaptive mutations in E2 that allowed the virus to utilize widely expressed cell surface heparan sulfate (HS) as an attachment receptor, thereby increasing infectivity in cultured cells [47]. Although this HS adapted strain showed reduced virulence, it still elicited robust humoral and cellular immune responses in animals, generating high levels of neutralizing antibodies [47], suggesting a viable strategy for designing broad spectrum arbovirus vaccines. Genome wide CRISPR Cas9 screens further indicate that GAG biosynthetic enzymes (e.g. B3GAT3, SLC35B2) influence CHIKV binding [37].

#### Prohibitin (PHB)

PHB is a multifunctional membrane protein involved in mitochondrial function, cell proliferation, and apoptosis. Using virus overlay protein binding assays, Phitchayapak et al. [48] identified PHB as a primary CHIKV binding protein on host cells. Pretreatment with anti PHB antibodies or knockdown of PHB expression reduced CHIKV infection, whereas PHB overexpression rendered non permissive cells susceptible. Co immunoprecipitation confirmed direct interaction between CHIKV E2 and PHB. Moreover, flavaglines, plant compounds that bind PHB, effectively inhibit CHIKV entry in cellular models [49].

#### Dendritic cell-specific intercellular adhesion molecule-3-grabbing non-integrin (DC-SIGN/L-SIGN)

DC-SIGN (CD209) and its homolog L-SIGN (CD209L) are C-type lectin receptors that function as attachment factors for multiple alphaviruses, including CHIKV [50]. However, some studies suggest that DC-SIGN/L-SIGN functions solely as an attachment factor for SINV and does not facilitate CHIKV entry [51]. DC-SIGN is primarily expressed on immature dendritic cells (DCs) and macrophage subpopulations present in the dermis, mucosal surfaces, and lymph nodes [52]. L-SIGN is expressed on liver sinusoidal endothelial cells and lymph node endothelia [52]. As key pattern recognition receptors, DC-SIGN mediates the initial attachment and internalization of CHIKV in DCs through high-affinity binding to the high-mannose glycans on the viral E2 glycoprotein [52,53]. Studies have confirmed that parental 3T3 cells lacking DC-SIGN are not susceptible to CHIKV, while the overexpression of DC-SIGN significantly enhances their susceptibility to CHIKV [53]. Unlike MXRA8 which mediates productive infection in fibroblasts, engagement of DC-SIGN during viral entry may also modulate innate immune responses, potentially influencing the balance between viral clearance and immune-mediated pathology [53], which is a key link between viral infection and host inflammatory response.

#### Toll-like receptor 4 (TLR4)

TLR4 is a key pattern recognition receptor of the innate immune system, mainly expressed in macrophages, monocytes, epithelial and endothelial cells [54]. Recent studies have found that TLR4 not only mediates the recognition of CHIKV pathogen-associated molecular patterns (PAMPs) to trigger antiviral immune response but also directly participates in the viral entry process [54,55]. The viral E2 glycoprotein can directly bind to the extracellular domain of TLR4, which promotes the attachment and endocytosis of CHIKV in macrophages [55]. Blocking TLR4 with specific inhibitors (such as TAK-242) can significantly reduce CHIKV entry and pro-inflammatory responses in mouse and human macrophages [55]. In addition, TLR4-mediated signaling activation is a core driver of the inflammatory storm during acute CHIKV infection, which links viral entry to the subsequent inflammatory pathological process [56,57].

CHIKV exhibits striking cell type specificity in the host factors. It exploits for cellular attachment and productive infection, with distinct repertoires of receptors and attachment factors governing viral entry across its diverse target cell populations. The canonical functional receptor MXRA8 serves as the primary mediator of CHIKV infection in mesenchymal lineage cells including fibroblasts, osteoblasts, chondrocytes, and skeletal muscle cells, while non-MXRA8-expressing cells rely on fully distinct, cell-restricted host factors: macrophages and renal epithelial cells depend on the phosphatidylserine receptors TIM-1 and Axl, epithelial cells utilize GAGs and PHB as key determinants of infection efficiency, and dendritic cells exclusively employ DC-SIGN to mediate viral attachment and internalization. This divergent, cell-specific utilization of host entry factors not only expands the systemic tissue tropism of CHIKV by enabling infection of MXRA8-negative cell types and complementing the canonical MXRA8-mediated entry pathway, but also links viral entry to cell-type-specific host immune responses, as exemplified by TLR4’s dual role in mediating both CHIKV entry and inflammatory activation in macrophages. Taken together, CHIKV exploits a variety of host factors to invade diverse target cells, which endows it with broad tissue tropism and thus places higher demands on the prevention and control of this virus.

### Host factors affecting CHIKV RNA replication

During replication, CHIKV nsPs assemble with multiple host factors to form vRCs, where negative strand RNA is synthesized using the genomic RNA as a template, generating a dsRNA intermediate for efficient amplification. Key host factors involved in this phase include four and a half LIM domain protein 1 (FHL1), cluster of differentiation 81 (CD81), GTPase activating protein (SH3 domain) binding proteins 1 and 2 (G3BP1/2), bridging integrator 1 (BIN1), musashi RNA binding protein 2 (MSI2), receptor for activated C kinase 1 (RACK1), DEAD box helicase 56 (DDX56), sphingosine kinase 2 (SK2), nucleophosmin (NPM1/B23), and Y box binding protein 1 (YBX1).

#### Four and a half LIM domain protein 1 (FHL1)

FHL1 is highly expressed in skeletal and cardiac muscle, containing four half-LIM domains. Among its three splice variants, FHL1A is present in muscle, fibroblasts, and other tissues. A genome wide CRISPR Cas9 screen identified FHL1 as essential for CHIKV infection, and FHL1A knockout increased susceptibility to CHIKV and ONNV [22]. Further studies showed that FHL1A interacts with CHIKV nsP3 to regulate RNA replication. FHL1A deletion impaired negative strand RNA synthesis and vRC formation, findings validated in human primary cells and mouse models [22]. Elevated serum FHL1 levels in acute and chronic CHIKF patients further suggest its role in infection and disease progression [58].

#### Cluster of differentiation 81 (CD81)

CD81, a tetraspanin family protein involved in cell adhesion and signaling, promotes CHIKV RNA replication by colocalizing with viral dsRNA [59]. Although CD81 facilitates entry of other viruses (e.g. HCV), it does not affect CHIKV entry. Multiple alphaviruses, including RRV, SFV, SINV, and VEEV, also require CD81 for infection. CD81’s proviral function is linked to cholesterol binding, indicating that alphaviruses hijack this cholesterol interacting membrane microdomain protein to support replication [59].

#### Gtpase-activating protein (SH3 domain)-binding protein (G3BP1/G3BP2)

Stress granules (SGs) are cytoplasmic aggregates of proteins and untranslated mRNAs that form under cellular stress and generally exert antiviral effects [60]. G3BP1 is a key SG component and marker, while its homolog G3BP2 is less characterized. CHIKV infection induces cytoplasmic granules containing G3BP1 and G3BP2. Simultaneous knockdown of both proteins reduces CHIKV RNA levels, protein expression, and progeny titers, indicating that CHIKV coopts G3BP proteins for efficient replication [61]. Cryo-EM studies show that G3BP1 binds CHIKV nsP3, promoting infection by suppressing SG formation and interfering with interferon responses [62].

#### Bridging integrating factor 1 (BIN1)

BIN1 participates in membrane remodeling and endocytosis. Its SH3 domain binds directly to the hypervariable domain (HVD) of CHIKV nsP3 and that of other alphaviruses [63]. This interaction has been observed in mosquito C6/36 cells, human HuH-7 cells, and mouse NIH/3T3 cells but is absent in HOS and HEK293 cells, indicating cell type specificity [63,64].

#### Musashi RNA-binding protein-2 (MSI2)

MSI proteins are conserved RNA binding proteins containing two RNA recognition motifs. MSI2 deficiency or small molecule inhibition significantly impairs CHIKV genome replication [65]. MSI2 specifically binds the sequence 63 AUUAAU 68 within the CHIKV 5’ UTR. Electrophoretic mobility shift assays confirmed this interaction, and mutation of the site (to 63 CAACUU 68) disrupted MSI2 binding, inhibiting replication and preventing recovery of mutant virus [65]. The small molecule inhibitor Ro 08 2750 (which targets both MSI1 and MSI2) significantly reduces CHIKV genomic replication. MSI1 also exhibits proviral effects in multiple cell lines.

#### Scaffold protein receptor for activated C kinase 1 (RACK1)

RACK1, initially identified as a protein kinase C anchor, participates in diverse cellular processes and has been shown to promote viral infection through multiple mechanisms [66]. Recently, RACK1 was identified as a novel host interactor and regulator of CHIKV nsP4, protecting nsP4 from ubiquitin proteasome mediated degradation [66]. Co-IP and GST pull down assays confirmed that RACK1 binds directly to the RdRp domain of nsP4. Disruption of this interaction with the RACK1 inhibitor harringtonolide decreased nsP4 protein levels, and cycloheximide chase experiments showed that RACK1 stabilizes nsP4. Proteasome inhibitor MG132 reversed harringtonolide induced nsP4 degradation, confirming that RACK1 protects nsP4 from proteasomal degradation [66].

#### DEAD box helicase 56 (DDX56)

DDX56 is a nucleolar helicase involved in ribosome biogenesis. During CHIKV infection, DDX56 relocalizes to the cytoplasm and binds directly to viral genomic RNA via a conserved stem loop structure adjacent to the RdRp start codon, a region critical for efficient translation and infection initiation [67,68] DDX56 knockdown did not affect poly(I:C) induced interferon or interferon stimulated gene expression, indicating an interferon independent antiviral mechanism [67].

#### Sphingosine kinase 2 (SK2)

Sphingosine kinases (SK1 and SK2) catalyze the production of sphingosine 1 phosphate (S1P) [69,70]. SK2, but not SK1, positively regulates CHIKV replication [69]. siRNA mediated SK2 knockdown or treatment with the specific inhibitor ABC294640 reduced CHIKV protein expression and RNA release in a dose dependent manner. Upon infection, SK2 redistributes from the nucleus to cytoplasmic puncta that colocalize with viral dsRNA, nsP2, and newly synthesized RNA in vRCs. SK2 also co purifies with nsP3 and G3BP1, suggesting nsP3 recruits SK2 to replication sites. AP MS analysis indicated that CHIKV infection induces SK2 to complex with host proteins involved in mRNA processing and gene expression, implicating SK2 in reprogramming host gene expression to support viral replication [69].

#### Nucleophosmin (NPM1/B23)

NPM1/B23 acts as a host restriction factor against CHIKV through dual mechanisms: direct interaction with viral proteins and modulation of innate immunity [71,72]. During infection, NPM1 shifts from the nucleolus to the cytoplasm, where it accumulates at later stages. Artificially retaining NPM1 in the nucleus abolishes its antiviral effect, whereas a constitutively cytoplasmic NPM1 variant suppresses infection. NPM1 enhances the expression of interferon stimulated genes (IRF1, IRF7, OAS3) and directly binds the macrodomain of nsP3, potentially interfering with nsP3 function. This interaction depends on residues N24 and Y114 within the nsP3 macrodomain, which are also essential for its ADP ribosylhydrolase activity [71].

#### Y-Box binding protein 1 (YBX1)

A genome wide CRISPR/Cas9 screen identified YBX1 as essential for the replication of multiple alphaviruses [73]. YBX1 does not affect viral entry but relocalizes to vRCs post entry, where it interacts with nsP3 and viral RNA. YBX1 enhances the RNA binding capacity of nsP3, promoting vRC assembly and RNA replication. Mutations in the YBX1 RNA binding domain or the YBX1 binding motif on nsP3 severely impair viral replication. YBX1 deficiency inhibits replication of all tested Old and New World alphaviruses without affecting unrelated viruses, indicating broad spectrum alphavirus specific activity [73].

### Host factors affecting CHIKV RNA translation

In addition to replication factors, DExH box helicase 9 (DHX9) plays a role in translational regulation. DHX9 is an NTP dependent helicase involved in transcription, splicing, and translation. It interacts with nsP2 and nsP3 of CHIKV and other alphaviruses, promoting the translation of viral genomic RNA [74–76].

### Host factors affecting CHIKV assembly and release

Alphaviruses rely on the host cell transport system to transport its envelope proteins to the plasma membrane. siRNA screening indicates that multiple host factors in the actin remodeling pathway participate in this process [77]. Actin rearrangement occurs during late infection, and E2 colocalizes with actin filaments. ADP ribosylation factor 1 (Arf1) and Ras related C3 botulinum toxin substrate 1 (Rac1) contribute to the formation of E2/E1 containing transport vesicles, which move along actin filaments via the Rac1/Arp3/PIP5K1α pathway to mediate viral budding [77].

**Figure 2.**
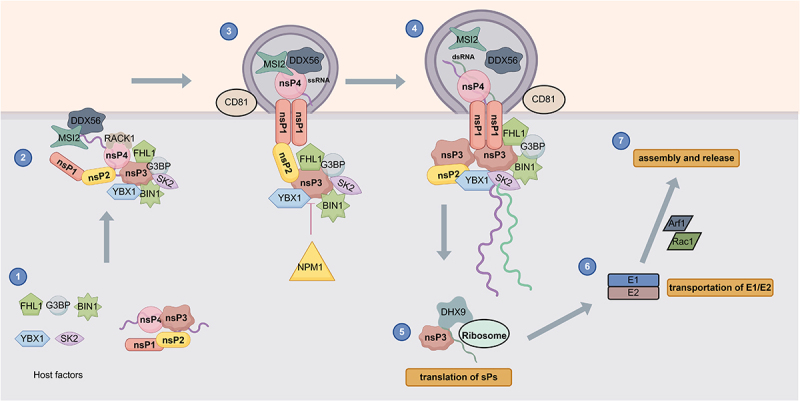
Model of CHIKV replication complex assembly and host factors affecting CHIKV RNA replication.

CHIKV depends on the coordinated assembly of viral non-structural proteins and a wide spectrum of host factors to form functional vRCs, with each host factor exerting highly specialized, non-redundant roles in vRC formation, stabilization, and catalytic function throughout the replication process. The hypervariable domain of nsP3 serves as the central interaction hub for vRC assembly, directly recruiting host factors including FHL1, G3BP1/2, BIN1, YBX1, and SK2; these recruited factors fulfill discrete, essential functions to support vRC activity, with FHL1 governing negative-strand RNA synthesis and de novo vRC formation, G3BP1/2 suppressing antiviral stress granule formation to maintain vRC integrity, BIN1 mediating membrane remodeling for vRC anchoring in a cell-type-specific manner, YBX1 enhancing nsP3’s RNA-binding capacity to promote vRC assembly, and SK2 reprogramming the local host protein network to sustain efficient viral replication. Additional host factors further modulate vRC function through distinct, non-overlapping mechanisms. CD81 stabilizes the cholesterol-rich membrane microenvironment of vRCs. MSI2 binds the viral 5’ UTR to initiate genome replication. RACK1 protects the viral RNA-dependent RNA polymerase nsP4 from proteasomal degradation to preserve the catalytic core of vRCs. And, DDX56 binds viral genomic RNA to facilitate replication initiation. While, NPM1 acts as a key host restriction factor that translocates to the cytoplasm to disrupt nsP3 function and boost antiviral innate immunity, negatively regulating vRC activity at late stages of infection. Beyond regulating vRC-mediated RNA replication, this intricate host factor interaction network is tightly coupled to subsequent steps of the CHIKV life cycle, with DHX9 governing viral RNA translation and actin remodeling factors such as Arf1 and Rac1 mediating envelope protein trafficking and progeny virion assembly and release, collectively defining a comprehensive regulatory network that dictates CHIKV infection efficiency and offers promising broad-spectrum therapeutic targets against alphaviruses (Figure 2).

## Immune signaling pathways associated with CHIKV infection and related chronic arthritis

CHIKV infection poses a significant global public health burden, with its most debilitating sequela being chronic inflammatory arthritis, where the host innate immune system acts as both the first line of antiviral defense and the critical driver of inflammatory cascade activation, infection outcome, and chronic arthritis progression. In this section, we delineate the two core pattern recognition receptor signaling axes responsible for CHIKV sensing: the RLRs-MAVS pathway and TLRs-mediated cascades. Then, we further elaborate on key host cellular signaling pathways reprogrammed by CHIKV, including JAK/STAT, MAPK, and PI3K/Akt/mTOR pathways, which function as central hubs of antiviral immunity.

### Main signaling pathways for innate immune sensing and inflammation triggering in CHIKV infection

Innate immunity serves as the first line of host defense against CHIKV infection, and acts as the critical initiating link that drives the onset of the inflammatory cascade, dictates infection outcomes, and governs the development of chronic arthritis. Upon host entry, CHIKV initially infects local dermal fibroblasts, macrophages, endothelial cells, and keratinocytes within the skin, before disseminating via the bloodstream to target organs including the joints and skeletal muscle [2,78]. Viral genomic RNA and replication intermediates are subsequently recognized by host pattern recognition receptors (PRRs), triggering the activation of downstream core signaling pathways [79].

#### RLRs-MAVS signaling pathway

Retinoic acid-inducible gene I (RIG-I)-like receptors (RLRs) are the core receptor family responsible for recognizing intracellular RNA of CHIKV [80], which consists of three members: RIG-I, MDA5, and LGP2. Among them, RIG-I specifically recognizes the 3’ UTR of the CHIKV genome, while MDA5 recognizes dsRNA generated during viral replication [81,82]. Upon activation, both transduce signals via the mitochondrial antiviral signaling protein (MAVS) to trigger downstream signaling cascades [83]. On one hand, they activate Interferon Regulatory Factor 3/7 (IRF3/7) to induce the expression of Type I interferons (IFN-Is, including IFN-α and IFN-β), thus initiating the IFN-JAK/STAT-interferon-stimulated gene (ISG) antiviral axis [84,85]. On the other hand, they activate Nuclear Factor κB (NF-κB) to drive the transcription and release of pro-inflammatory cytokines [86,87]. The functional status of the RLRs signaling pathway directly dictates the outcome of CHIKV infection. Activation of RIG-I and MDA5 effectively restricts viral replication, whereas LGP2, as a negative regulatory factor of the RLRs family, can interfere with RIG-I-mediated immune responses [88].

#### Tlrs-mediated signaling pathway

Within the Toll-like receptor (TLR) family, TLR3 and TLR7/8 are the members involved in CHIKV recognition and inflammatory regulation. Both trigger downstream signaling through distinct adaptor proteins and are closely associated with the pathological progression of chronic arthritis.

#### TLR3-TRIF Signaling Pathway

TLR3 recognizes viral dsRNA and serves to protect the host against viral infection [89]. It activates the IRF3 and NF-κB pathways via the adaptor protein TRIF [90]. TLR3 deficiency results in defective host antiviral responses, markedly enhanced viral replication, impaired production of CHIKV-specific neutralizing antibodies, and aggravated pathological damage to joint tissues [91]. However, excessive and sustained activation of TLR3 drives the overrelease of inflammatory mediators such as IL-6 and IFNs, amplifies local inflammatory responses in the joints, and contributes to the progression of chronic arthritis [92].

#### TLR7/8-MyD88 Signaling Pathway

TLR7 and TLR8 are encoded on the X chromosome and specifically recognize viral single-stranded RNA (ssRNA) [93]. They activate the NF-κB and mitogen-activated protein kinase (MAPK) pathways through myeloid differentiation factor 88 (MyD88) [94]. This pathway exerts antiviral effects by inducing the formation of neutrophil extracellular traps (NETs) and the release of pro-inflammatory cytokines [95]. Meanwhile, TLR7/8 gene polymorphisms are directly associated with susceptibility to CHIKV infection [96]. In addition, the sex-differential expression of the TLR7/8 pathway is also recognized as an important contributor to the sex-based disparities in CHIKV infection and arthritis incidence during epidemics [97].

### Main immune signaling pathways mediating CHIKV-induced inflammatory responses and related chronic arthritis

CHIKV infection can reprogram multiple host cellular signaling pathways, which serve not only as the regulatory hubs of host antiviral immunity, but also as the targets exploited by the virus to achieve immune evasion, drive inflammatory amplification, and promote the onset and progression of chronic arthritis.

#### JAK/STAT signaling pathway

The JAK/STAT pathway is an important pathway for IFN-I-mediated antiviral immunity, and a primary target for CHIKV-induced immune evasion and inflammatory dysregulation [98]. Following receptor binding, IFN-Is activate JAK1/2 kinases, promote STAT1/2 phosphorylation and heterodimer formation, which translocate to the nucleus to trigger ISG transcription. This induces the expression of antiviral proteins such as IFITs, OAS3, PKR, BST2 and Viperin (RSAD2), thus limiting viral replication. CHIKV potently antagonizes this pathway through its non-structural protein nsP2. nsP2 directly blocks STAT1 nuclear translocation, disrupts STAT1/STAT2 complex formation, and globally suppresses JAK/STAT signal transduction [98]. This not only abrogates the antiviral activity of IFN-Is to facilitate persistent viral infection but also causes dysregulation of host anti-inflammatory immunity. Notably, IL-27 dysfunction is the central mechanism linking aberrant JAK/STAT signaling to chronic arthritis [99]. However, nsP2-mediated JAK/STAT inhibition eliminates the antiviral and anti-inflammatory functions of IL-27 [100], while retaining its pro-inflammatory activity, which continuously drives joint inflammation and pain and ultimately leads to chronic arthritis.

#### MAPK signaling pathway

The mitogen-activated protein kinase (MAPK) family constitutes the signaling axis driving inflammatory amplification, viral replication, and joint tissue damage during CHIKV infection. It comprises three major branches: p38, JNK, and ERK, among which p38 and JNK are the vital targets mediating the onset and progression of chronic arthritis.

#### p38 MAPK signaling pathway

The CHIKV nsP2 protein directly engages in polar interaction with p38, and mediates the phosphorylation and activation of p38 via transforming growth factor-β-activated kinase 1 (TAK1) [101]. Activated p38 acts through its downstream effector molecules MK2/3 to exert dual biological functions [102]. On one side, it promotes viral replication and progeny virion assembly; on the other side, it directly drives robust transcription and release of pro-inflammatory cytokines including TNF-α, IL-1β, and IL-6, which induces the activation of joint synovial fibroblasts and cartilage matrix degradation. This pathway is therefore a driver of joint inflammation and structural damage.

#### JNK signaling pathway

CHIKV nsP2 can directly bind to the phosphorylation domain of JNK and activate this pathway [101,103]. JNK phosphorylates and activates the downstream transcription factor c-Jun, thereby directly driving the transcriptional expression of TNF. JNK inhibitors significantly downregulate phosphorylated c-Jun (p-c-Jun) expression in a dose-dependent manner, whereas p38 inhibitors exert no significant regulatory effect on p-c-Jun expression [101], which definitively identifies the JNK/p-c-Jun/TNF axis as an important pathway mediating the inflammatory response following CHIKV infection. The JNK pathway, together with the p38 pathway, co-regulates the phosphorylation and activation of the transcription factor IRF3 to participate in the host innate antiviral immune response. Meanwhile, the JNK pathway promotes TCR-driven T cell activation by inducing TNF production [101], further amplifying local and systemic inflammatory responses, and is involved in the regulation of chronic pathological processes including arthritis following CHIKV infection.

#### ERK signaling pathway

Autophagy induced by CHIKV infection cleaves and downregulates ERK1/2, thus inhibiting the RAS-RAF-MEK-ERK pathway [100,104]. This results in the blockade of host cap-dependent translation, while the virus achieves preferential translation of its own proteins via the Mnk/eIF4E pathway, ultimately causing host cell dysfunction [104,105].

#### PI3K/Akt/mTOR signaling pathway

The phosphatidylinositol 3-kinase-Akt-mTOR (PI3K/Akt/mTOR) pathway exerts cell type-specific bidirectional regulatory effects during CHIKV infection [100]. It is not only involved in the regulation of viral replication and immune evasion but also mediates inflammatory responses and cell survival, and is closely associated with persistent viral infection and the onset and progression of chronic arthritis. In nonimmune cells, such as fibroblasts, CHIKV activates the PI3K/Akt/mTOR pathway via nsP3 [39], which exerts a potent anti-apoptotic effect, prolongs host cell survival, and provides a stable cellular niche for sustained viral replication [106]. This leads to persistent infection of joint tissues, and forms a critical pathological basis for the persistence of chronic inflammation. Whereas in immune cells such as macrophages, activation of the PI3K/Akt pathway upregulates the expression of interferon-stimulated genes (ISGs) and pro-inflammatory cytokines through the mTOR/p70S6K axis, further amplifying local inflammatory responses in the joints [100,107]. Meanwhile, CHIKV can inhibit the activity of mammalian target of rapamycin complex 1 (mTORC1) to evade the host translational repression machinery, and enables preferential translation of its own proteins via the PI3K-Mnk-eIF4E pathway [105]. This persistently disrupts the normal physiological functions of host cells and drives sustained release of inflammatory mediators.

### Other regulatory pathways associated with chronic arthritis

#### Wnt/β-catenin signaling pathway

CHIKV nsP2 suppresses β-catenin activation, upregulates glycogen synthase kinase-3β (GSK-3β) and downregulates cyclin D1 expression, triggering cell cycle dysregulation and impaired proliferation and repair of joint tissue cells [108], which contributes to chronic joint tissue damage. Studies have confirmed that the β-catenin inhibitor iCRT14 reduces viral load by 91.7% in CHIKV-infected mice and significantly alleviates disease symptoms [108], verifying the important role of this pathway in CHIKV infection and arthritis.

#### TGF-β signaling pathway

Bidirectional dysregulation of the TGF-β pathway plays a critical role in CHIKV-induced chronic arthritis. In acute CHIKV infection, regulatory T cell (Treg) function is inhibited and TGF-β levels are significantly downregulated, weakening host anti-inflammatory capacity and leading to uncontrolled excessive inflammation [109]. In the chronic phase, however, aberrantly upregulated TGF-β mediates joint synovial fibrosis and abnormal tissue remodeling, aggravating joint stiffness and dysfunction [110,111].

#### DNA Damage Response (DDR) signaling pathway

Multiple in vitro, in vivo and ex vivo models verified that CHIKV infection activates the core ATM/ATR-Chk1/Chk2 DDR pathway [112]. CHIKV non-structural protein nsP2, as a key regulatory molecule, directly interacts with Chk1 and Chk2. Activation of the DDR pathway induces dual G_1_/G_2_ cell cycle arrest, which establishes a stable cellular microenvironment for efficient viral replication. It also modulates the cytokine network by upregulating pro-inflammatory factors and inhibiting IFN-β, thus impairing the host antiviral immune response. Silencing of Chk1/Chk2 or inhibition of ATM/ATR by the ATM/ATR kinase inhibitor (AAKi) drastically reduced viral load (up to 99.7%) and alleviated infection-associated symptoms [112]. These findings confirm that the DDR pathway is an essential host factor for CHIKV replication and identify it as a promising therapeutic target for anti-CHIKV interventions.

**Figure 3.**
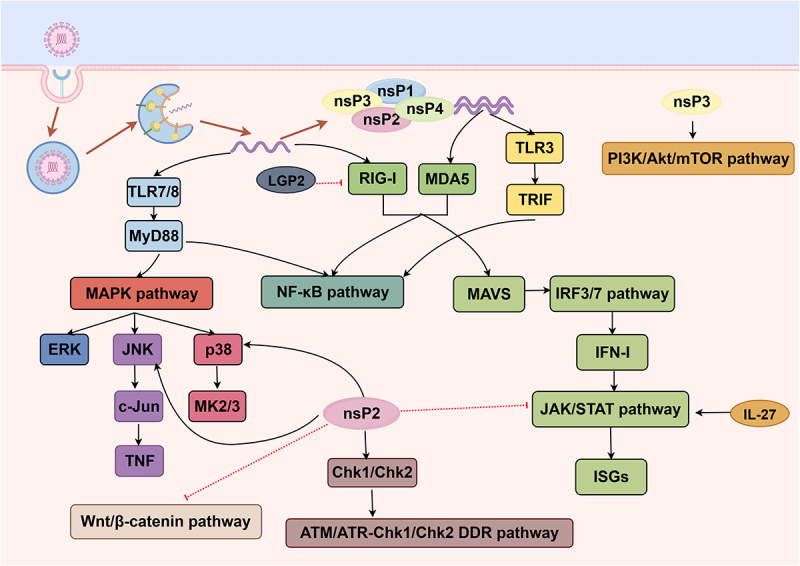
Immune signaling pathways associated with CHIKV infection.

The RLRs-MAVS and TLRs-mediated pattern recognition pathways act as complementary upstream sensors for CHIKV infection, converging on the core transcription factors IRF3/7 and NF-κB to initiate type I interferon (IFN-I)-mediated antiviral immunity and pro-inflammatory cytokine production, while forming a feedforward amplification loop with the downstream JAK/STAT axis to shape the host’s initial antiviral defense. CHIKV, predominantly via its nsP2 and nsP3, extensively reprograms this integrated signaling network, driving complex bidirectional crosstalk between the MAPK (p38, JNK), PI3K/Akt/mTOR, and auxiliary regulatory pathways (including Wnt/β-catenin, TGF-β, and DDR signaling), which simultaneously enables viral immune evasion and persistent replication while amplifying and sustaining inflammatory responses in joint tissues. The coordinated physiological activation of these interconnected pathways supports effective viral clearance and resolution of infection, whereas CHIKV-induced multi-node dysregulation of the signaling network impairs host antiviral immunity, drives unabated inflammatory amplification, and ultimately promotes the onset and progression of debilitating CHIKV-associated chronic arthritis (Figure 3).

## Research progress on anti-CHIKV strategies

No specific antiviral drugs are currently approved for CHIKV; clinical care is limited to symptom relief, underscoring the urgent need for effective therapies [18]. Drug development efforts focus on two main strategies: directly targeting viral proteins or targeting host factors essential for viral replication. Given the high mutation rate of CHIKV and the risk of drug resistance associated with direct-acting antivirals (DAAs), host-targeted antivirals (HTAs) have become a research hotspot in recent years. This section focuses on the research progress of HTAs, and only retains the DAA content related to host factors, while systematically evaluating the druggability, benefit-risk profile and clinical translation potential of different host targets.

### Anti-CHIKV drugs targeting host factors

Although there are some progresses in drug research targeting CHIKV structural proteins and nonstructural proteins [18], drug resistance caused by viral mutations should be concerned. Consequently, HTAs have garnered increasing attention. Compared to RNA viral genomes, HTAs offer advantages of higher genetic stability and potentially broader antiviral spectra, reducing the risk of resistance. The following are the advances on the anti-CHIKV HTAs (Supplementary table 2).

#### Host factors inhibitors targeting CHIKV entry

Verma et al. [113] identified three compounds (ZINC299817498, ZINC584908978, and LAS52155651) through molecular dynamics simulations and binding free energy calculations. These compounds stably bind to the E1-E2-MXRA8 complex interface and possess the potential to block this interaction. Monoclonal antibodies targeting this receptor (e.g. CHK-124) can effectively compete for MXRA8 binding, thereby blocking viral receptor engagement [114]. Natural plant compounds flavaglines (FL) and their synthetic analogues (FL3 and FL23), along with sulfonamides, all bind to PHB1 [115,116], exhibiting potential antiviral effects. Pre-treatment of cells with FL3 and FL23 prior to infection significantly inhibited CHIKV entry, and immunofluorescence revealed reduced colocalization of PHB1 with the viral E2 protein; however, post-infection administration showed no inhibitory effect, indicating their activity is restricted to the entry phase [49]. Compounds such as chebulagic acid and punicalagin block viral glycoprotein-GAG interactions within an effective concentration range of 10–100 μmol/L, thereby blocking viral entry [117]. However, the druggability and safety of heparan sulfate mimetics require further evaluation due to GAGs’ broad cellular roles.

#### Host factors inhibitors targeting viral replication

A large number of host factors involved in CHIKV replication have become potential therapeutic targets. The MSI2 inhibitor Ro 08–2750 can significantly inhibit the replication of CHIKV in vitro [65]. The RACK1 inhibitor Harringtonolide can promote the proteasomal degradation of nsP4 [66]. The SK2 specific inhibitor ABC294640 has completed phase I clinical trials for the treatment of solid tumors [118], with clear safety and pharmacokinetic data in humans, and has shown good anti-CHIKV activity in preclinical studies, which has high repurposing potential [69].

#### Inhibitors targeting immune and inflammatory signaling pathways

The excessive inflammatory cascade induced by CHIKV infection constitutes the central mechanistic driver of tissue injury, organ damage, and the progression to chronic disease in infected hosts [119,120], and targeting the key host factors of inflammatory signaling pathways has become an important strategy for the treatment of CHIKF, especially for chronic persistent arthralgia [5]. The TLR4 inhibitor TAK-242 has completed phase III clinical trials for the treatment of sepsis [121], and preclinical studies have confirmed that it can inhibit the entry of CHIKV in macrophages and synovial cells [55]. In addition, TAK-242 can block the activation of NF-κB signaling, significantly reducing the secretion of pro-inflammatory cytokines and alleviating joint inflammation in rat experimental autoimmune neuritis [122], which suggesting that it may alleviate the inflammatory response induced by CHIKV infection through this pathway [55]. The JAK inhibitor baricitinib, which has been approved for the treatment of rheumatoid arthritis, can inhibit the JAK-STAT signaling pathway, reduce the inflammatory response [123]. SB203580, a specific p38 MAPK inhibitor, significantly suppresses TNF-α production and the inflammatory response induced by CHIKV infection in macrophages, without obviously affecting viral replication, by blocking the p38 signaling pathway activated through interaction with CHIKV nsP2 [101]. AKT inhibitors (AKKi) suppress AKT activation and the downstream DDR signaling induced by CHIKV infection, thereby significantly impairing viral genome replication and progeny production [112]. In addition, the anti-TNF-α monoclonal antibody adalimumab and the anti-IL-6 receptor monoclonal antibody tocilizumab, which are widely used in the treatment of rheumatoid arthritis, have shown good efficacy in the treatment of chronic CHIKV-related arthralgia in clinical case reports and small-scale clinical trials, which can significantly alleviate joint symptoms and improve the quality of life of patients [124].

#### Other antiviral drugs targeting signaling pathways

Hamucurine and analogues (tigubiflorine, desoxy-tigubiflorine) inhibit CHIKV non-structural proteins translation by blocking eukaryotic translation initiation factor 2α, thereby suppressing viral RNA synthesis and structural protein expression [125]. This mechanism also underlies their broad-spectrum activity against EBV and influenza viruses, with no reported viral resistance. A systematic siRNA/drug repurposing screen identified 20 compounds targeting six host pathways. The fatty acid synthase inhibitor TOFA combined with the calmodulin inhibitor pimozide synergistically reduced CHIKV replication and joint swelling in mice [126]. Lipid metabolism is critical for CHIKV membrane fusion. Inhibitors of fatty acid synthase, stearoyl CoA desaturase 1 (orlistat, azulen, CAY10566), and cholesterol transport (imipramine, U18666A) suppress CHIKV replication [127,128].

At present, drugs targeting host factors for CHIKV chronic arthritis, including NSAIDs, SAIDs, DMARDs and colchicine, have been used in clinical. NSAIDs and SAIDs exert anti-inflammatory effects by inhibiting inflammatory mediator synthesis and systemic immune activation, respectively, methotrexate suppresses the activation of inflammatory cells in synovial tissue, and colchicine blocks neutrophil migration and intracellular signaling, while chloroquine and hydroxychloroquine show no benefit as monotherapy [129]. Targeting inflammatory signaling host factors does not directly affect viral replication, so there is no risk of inducing drug-resistant strains. And, they can alleviate the clinical symptoms caused by excessive inflammatory response, and are suitable for the treatment of both acute severe CHIKF and chronic persistent arthralgia. Most of the related drugs are approved for the treatment of rheumatoid arthritis and other inflammatory diseases, with clear human safety, efficacy and pharmacokinetic data, and have direct clinical application value. While, there are still multiple limitations. They cannot inhibit viral replication, so they need to be used in combination with antiviral drugs in the acute infection stage; Long-term use of immunosuppressive drugs may increase the risk of secondary infection, and the benefit-risk ratio needs to be strictly evaluated in clinical application.

### Direct-acting antiviral drugs targeting CHIKV proteins (only retain content related to host factors)

Direct-acting antivirals target the conserved functional domains of CHIKV structural and non-structural proteins and have the advantages of high specificity and strong antiviral activity. However, the high mutation rate of CHIKV (RNA virus with a mutation rate of ~10^−4^ substitutions per site per replication cycle) can easily lead to the emergence of drug-resistant strains, which limits the long-term efficacy of single DAA therapy. It is worth noting that some DAAs can also exert antiviral effects by regulating host factors or signaling pathways. For example, the nsP2 protease inhibitor telmisartan can not only directly inhibit the protease activity of nsP2 but also regulate the host AT1/PPAR-γ/MAPKs signaling pathway to inhibit the inflammatory response caused by CHIKV infection [130]. And, the nsP4 RdRp inhibitor ribavirin can not only inhibit the activity of viral RdRp but also regulate the host innate immune response to enhance the antiviral effect [131]. The three synthesized IBU conjugates, IBU-SULFA, IBU-ISS, and IBU-IBT, generated by conjugating ibuprofen with sulfonamide and thiosemicarbazide pharmacophores, exert significant in vitro anti-CHIKV activity in Vero cells with favorable selectivity indices (all >10) predominantly by interfering with the early stages of the CHIKV life cycle (in contrast to the parent ibuprofen, which has no direct anti-CHIKV effect), exhibit no acute oral toxicity, retain and even enhance the anti-inflammatory and anti-arthritic properties of IBU in rat models while eliminating the ulcerogenic risk of native IBU, and therefore represent promising dual-action therapeutic candidates for simultaneously targeting CHIKV infection and its associated inflammatory and arthritic symptoms [132].

These dual-acting DAAs, which simultaneously target viral proteins and host pathways, have become an important direction for anti-CHIKV drug development. However, the limitations of these dual-targeting drugs are also obvious: their anti-CHIKV effective concentration in vitro is relatively high, and the oral bioavailability is low, which requires structural modification to improve their antiviral activity and pharmacokinetic properties. In addition, the clinical efficacy of these drugs in CHIKF patients still needs to be confirmed by large-scale randomized controlled trials.

### Combination strategies of DAAs and HTAs

Monotherapy of DAAs or HTAs for CHIKV, has critical inherent limitations. Accordingly, DAAs-HTAs combination therapy has emerged as the most promising anti-CHIKV therapeutic strategy. Robust preclinical evidence has validated multiple effective combination regimens. The combination of mefenamic acid and ribavirin exerts strong synergistic anti-CHIKV activity via complementary mechanisms (mefenamic acid blocks viral particle inactivation and host cell entry; ribavirin inhibits post-entry viral RNA replication). It reduced the in vitro EC_5__0_ to 3 μM (far below 13 μM for mefenamic acid alone and 10 μM for ribavirin alone), achieved a 6.5-fold in vivo reduction in blood viral titer, alleviated CHIKV-induced hepatosplenomegaly with no observable acute toxicity, and potently suppressed CHIKV infection both in vitro and in vivo [133]. The combination of doxycycline and ribavirin delivers synergistic and potent CHIKV inhibition by separately targeting the viral entry and replication stages of the CHIKV life cycle. This regimen significantly reduced the viral load in vitro and in vivo, mitigated infection-induced inflammatory pathological damage in the liver and spleen, improved the viability of CHIKV-infected cells, and exhibited favorable safety [134]. Dual-action candidate DDABT1 (telmisartan-salicylic acid ester conjugate) shows markedly enhanced in vitro anti-CHIKV activity (IC_5__0_=14.53 μM, selectivity index >33) by mainly targeting early-stage CHIKV infection. It outperforms single agents or their simple combination in treating CHIKV-related acute, subacute and chronic inflammation/arthritis in vivo, with excellent oral safety (LD_5__0_=5000 mg/kg in rats), making it a promising candidate for simultaneous antiviral and anti-inflammatory treatment [135]. Collectively, these preclinical findings strongly support the substantial translational potential of combination-based anti-CHIKV therapies. Future research priorities should focus on optimizing these combination regimens, and advancing large-scale clinical trials to validate their efficacy and safety in humans, which is critical to filling the unmet clinical need for effective CHIKV management.

In recent years, CHIKF outbreaks have increased in tropical and subtropical regions, posing a growing public health threat. Although research has greatly advanced our understanding of CHIKV biology and host virus interactions, no specific antiviral drugs are yet available, leaving clinicians dependent on supportive care. The roles of host factors as crucial facilitators of the viral lifecycle are increasingly recognized. However, most studies to date have focused on validating individual host factors, with limited exploration of systematic interaction networks and dynamic regulatory mechanisms. Moreover, the evolutionary interplay between viral variants and host immune responses remains unclear. Furthermore, the cell-type specificity of host factor exploitation by CHIKV and its implication in the transition from acute to chronic infection remain to be fully elucidated. In-depth analysis of the CHIKV-host interaction network has enriched our understanding of viral pathogenesis and opened new avenues for drug discovery.

Theoretically, any essential component of the viral replication cycle, including viral proteins and essential host factors, can serve as antiviral target. While significant progress has been made in developing direct acting antivirals against viral proteins, high mutation rates inevitably drive resistance. Targeting host factors offers a complementary, potentially more sustainable strategy, given their greater genetic stability and potential for broader antiviral spectra. However, this approach also faces challenges. Many host factors critical for viral replication also perform essential cellular functions, raising the risk of off target toxicity. Currently, most HTAs and novel direct acting compounds remain in preclinical validation, the translation of which to the clinic will require overcoming multiple pharmacological and safety hurdles. The key to the development of HTAs in the future is to identify host factors with high viral dependence and low physiological importance, so as to maximize the antiviral efficacy and minimize the on-target toxicity. In addition, the repurposing of approved drugs targeting host factors is an important way to accelerate the clinical translation of anti-CHIKV therapies. The combination of DAAs and HTAs, which simultaneously achieve efficient viral inhibition, resistance prevention, symptom relief and tissue protection, will become the mainstream direction of anti-CHIKV therapy development in the future.

## Supplementary Material

Supplemental Material

## Data Availability

Data sharing is not applicable to this article as no new data were created or analyzed in this study.
