# Supplementary material for: Research progress on the role of host factors in Chikungunya virus infection and intervention strategies
Source: Virulence. 2026 May 27;17(1):2679253. doi: 10.1080/21505594.2026.2679253 (PMC13217910; doi:10.1080/21505594.2026.2679253)
Supplement: Supplemental Material [file KVIR_A_2679253_SM7750.docx]

Supplementary table 1. Host factors affecting CHIKV infection

| Host factors | influencing stage | function | references |
| --- | --- | --- | --- |
| MXRA8 | entry | receptor | [32, 40] |
| PVEERs | entry | enhancing the binding and endocytosis | [41] |
| GAGs | entry | adhesion factor | [46] |
| PHB | entry | the primary binding protein | [48] |
| FHL1 | replication | interacting with CHIKV nsP3 to regulate CHIKV RNA replication | [22] |
| DC-SIGN/L-SIGN | entry/immune regulation | mediating viral attachment and internalization in DCs; regulating antigen presentation and innate immune response | [52, 53] |
| TLR4 | entry/immune/inflammatory regulation | mediating viral entry in myeloid cells and synovial cells; triggering innate immune and inflammatory signaling | [55, 57] |
| CD81 | replication | regulating CHIKV RNA replication by colocalizing with dsRNA | [59] |
| G3BP1/G3BP2 | replication | binding to CHIKV nsP3 | [62] |
| BIN1 | replication | binding to the HVD domain of CHIKV | [63] |
| MSI2 | replication | a key proviral factor for CHIKV replication | [65] |
| RACK1 | replication | a regulator of CHIKV nsP4 | [66] |
| DDX56 | replication | direct binding to CHIKV genomic RNA | [67, 68] |
| SK2 | replication | regulating the host gene expression machinery to assist viral replication | [69, 70] |
| NPM1/B23 | replication | directly binding to the macro domain of CHIKV nsP3 | [71, 72] |
| YBX1 | replication | enhancing the binding capacity of nsP3 to viral RNA | [73] |
| DHX9 | translation | binding to nsP2 and nsP3 | [76] |
| Arf1/Rac1 | assembly and release | participating in the formation of transport vesicles containing E2/E1 | [77] |

Supplementary table 2. Inhibitors targeting host factors

| inhibitor |  | host target/pathway | function | references |
| --- | --- | --- | --- | --- |
| ZINC299817498/ ZINC584908978/LAS52155651 |  | MXRA8 | binding to the E1-E2-MXRA8 complex interface | [113] |
| CHK-124 |  | MXRA8 | competing with the virus for binding to the MXRA8 receptor | [114] |
| FL3/FL23 |  | PHB | binding to PHB1; reducing the colocalization of PHB1 with the viral E2 protein | [115, 116] |
| chebulagic acid/punicalagin |  | GAGs | inhibiting viral glycoprotein-GAG interactions | [117] |
| Ro 08-2750 |  | MSI2 | inhibiting MSI2; blocking CHIKV genome replication | [65] |
| harringtonolide |  | RACK1 | disrupting the RACK1-nsP4 interaction; promoting nsP4 degradation; inhibiting viral replication | [66] |
| ABC294640 |  | SK2 | specifically inhibiting SK2; blocking CHIKV replication in vitro and in vivo | [69] |
| TAK-242 |  | TLR4/NF-κB | inhibiting TLR4 signaling; blocking viral entry and inflammatory response | [121, 122] |
| baricitinib |  | JAK-STAT | inhibiting JAK-STAT signaling; alleviating inflammatory joint damage | [123] |
| SB203580 |  | p38-MAPK | suppressing TNF-α production and the inflammatory response | [101] |
| AKT inhibitors (AKKi) |  | AKT | suppressing AKT activation and the downstream DDR signaling | [112] |
| adalimumab |  | TNF-α | anti-TNF-α monoclonal antibody | [124] |
| tocilizumab |  | IL-6 receptor | anti-IL-6 receptor monoclonal antibody | [124] |
| hamucurine/tigubiflorine/  desoxy-tigubiflorine |  | translation initiation | blocking eukaryotic translation initiation factor 2αsynthesis and RNA replication | [125] |
| TOFA |  | fatty acid synthesis | inhibiting host fatty acid synthesis; showing synergistic effect with Pimozide | [126] |
| Pimozide |  | calmodulin signaling | inhibiting calmodulin signaling; showing synergistic effect with TOFA | [126] |
| orlistat/azulen/CAY10566 |  | lipid metabolism | inhibiting FASN or SCD-1, disrupting membrane microdomains critical for CHIKV fusion and replication | [127] |
| Imipramine/U18666A |  | cholesterol transport | blocking CHIKV membrane fusion and replication | [128] |
| telmisartan |  | CHIKV nsP2/host AT1/PPAR-γ pathway | inhibiting nsP2 protease activity; regulating host inflammatory signaling | [130] |
